# Whole genome analysis of *Neisseria meningitidis* isolates from invasive meningococcal disease collected in the Czech Republic over 28 years (1993–2020)

**DOI:** 10.1371/journal.pone.0282971

**Published:** 2023-03-13

**Authors:** Michal Honskus, Pavla Krizova, Zuzana Okonji, Martin Musilek, Jana Kozakova

**Affiliations:** 1 National Reference Laboratory for Meningococcal Infections, Centre for Epidemiology and Microbiology, National Institute of Public Health, Prague, Czech Republic; 2 Third Faculty of Medicine, Charles University, Prague, Czech Republic; Cornell University, UNITED STATES

## Abstract

Invasive meningococcal disease belongs among the most dangerous infectious diseases in the world. Several polysaccharide conjugate vaccines against serogroups A, C, W and Y are available and two recombinant peptide vaccines against serogroup B (MenB vaccines) have been developed: MenB-4C (Bexsero) and MenB-fHbp (Trumenba). The aim of this study was to define the clonal composition of the *Neisseria meningitidis* population in the Czech Republic, to determine changes in this population over time and to estimate the theoretical coverage of isolates by MenB vaccines. This study presents the analysis of whole genome sequencing data of 369 Czech *N*. *meningitidis* isolates from invasive meningococcal disease covering 28 years. Serogroup B isolates (MenB) showed high heterogeneity and the most common clonal complexes were cc18, cc32, cc35, cc41/44, and cc269. Isolates of clonal complex cc11 were predominately serogroup C (MenC). The highest number of serogroup W isolates (MenW) belonged to clonal complex cc865, which we described as exclusive to the Czech Republic. Our study supports the theory that this cc865 subpopulation originated in the Czech Republic from MenB isolates by a capsule switching mechanism. A dominant clonal complex of serogroup Y isolates (MenY) was cc23, which formed two genetically quite distant subpopulations and which showed constant representation throughout the observed period. The theoretical coverage of isolates by two MenB vaccines was determined using the Meningococcal Deduced Vaccine Antigen Reactivity Index (MenDeVAR). Estimated Bexsero vaccine coverage was 70.6% (for MenB) and 62.2% (for MenC, W, Y). For Trumenba vaccine, estimated coverage was 74.6% (for MenB) and 65.7% (for MenC, W, Y). Our results demonstrated sufficient coverage of Czech heterogeneous population of *N*. *meningitidis* with MenB vaccines and, together with surveillance data on invasive meningococcal disease in the Czech Republic, were the basis for updating recommendations for vaccination against invasive meningococcal disease.

## Introduction

Invasive meningococcal disease (IMD) belongs among the most dangerous infectious diseases in the world. It is associated with high case fatality rate and a high percentage of severe lifelong sequelae in survivors. More than a million cases of IMD are reported worldwide each year, with an average fatality of 10–20%, but can reach up to 40% in cases caused by hypervirulent meningococcal clones. The vast majority of IMD cases are caused by six serogroups of *Neisseria meningitidis*: A, B, C, W, X, and Y [1,2].

Globally, experts agree that the best prevention of IMD is vaccination. Several polysaccharide conjugate vaccines against serogroups A, C, W, and Y are currently available [3]. Furthermore, two recombinant peptide vaccines against serogroup B (MenB vaccines) have been developed: MenB-4C (Bexsero) and MenB-fHbp (Trumenba) [4,5]. Since the genes encoding MenB vaccine antigens are present across isolates of all other serogroups, MenB vaccines have the potential to provide protection also against isolates of other serogroups (non-B). The theoretical coverage of non-B isolates of *N*. *meningitidis* with MenB vaccines represents an added value in vaccination programs [6] and is therefore currently being studied worldwide [7–10].

In the Czech Republic, vaccines which have been registered by the European Medicines Agency (EMA), are available for vaccination against IMD. These are three quadrivalent conjugate vaccines (MenACWY-TT2—Nimenrix, MenACWY-CRM—Menveo and MenACWY-TT—MenQuadfi) and both MenB vaccines (Bexsero, Trumenba). To achieve the highest possible protection against IMD, a combination of a conjugated ACWY vaccine and MenB vaccine is recommended in the Czech Republic. In accordance with Czech legislation, vaccination against IMD is covered by health insurance for patients with a medical indication (from January 2018), young children (from May 2020) and adolescents (from January 2022). Information about the vaccination strategy in the Czech Republic is available on the websites of the National Reference Laboratory for Meningococcal Infections (NRL) and the European Centre for Disease Prevention and Control [11,12].

Vaccination strategies in individual countries should be based on valid epidemiological data, including molecular characterization of the isolates that cause IMD. Whole genome sequencing (WGS) is the state-of-the-art method that provides wide opportunities to study the properties of *N*. *meningitidis*.

There are not many publications in the world that present the analysis of WGS data of *N*. *meningitidis* over a longer period. This paper presents an analysis of WGS data of 369 isolates from IMD from the Czech Republic for the period 1993–2020. The aim of this study was to define the clonal composition of the *N*. *meningitidis* population in the Czech Republic, to determine changes in this population over time and to estimate the theoretical coverage of isolates by MenB vaccines.

## Material and methods

### *N*. *meningitidis* isolates

In accordance with Czech legislation, *N*. *meningitidis* isolates from IMD are sent by laboratories from all over the country to the NRL for confirmation and further characterization. All received isolates are stored in the NRL collection lyophilized and/or frozen (-80 °C, Cryobank B, ITEST). For each isolate, clinical, epidemiological and microbiological data are available in the NRL database. The studied set consisted of a total of 369 isolates covering the 28-year period between 1993 and 2020, which was divided into four seven-year periods to enable the comparison of population trends of *N*. *meningitidis*: 1993–1999, 2000–2006, 2007–2013 and 2014–2020. The respective bacterial cultures were inoculated onto chocolate Mueller-Hinton agar and cultured at 37°C and 5% CO_2_ atmosphere for 18–24 hours. Correct identification of *N*. *meningitidis* was verified using the API NH kit (BIOMÉRIEUX). Serogroups were determined by standard serological methods (Pastorex Meningitis Bio-RAD, antisera *N*. *meningitidis* ITEST, Bio-RAD) and verified by the RT-PCR method [13].

### Whole genome sequencing and WGS data analysis

The QIAamp DNA Mini Kit (QIAGEN) was used for DNA isolation, and the isolation procedure was performed according to the manufacturer’s instructions. Whole genome sequencing was performed on the Illumina MiSeq platform and result was overlapping sequences of approximately 300 bp in length. The Velvet *de novo* Assembler software with Velvet-Optimiser script [14] was used to assemble the genomes from the primary raw data. The resulting genomes of individual isolates were submitted to the PubMLST database [15,16] and automatically characterized by the BIGSdb platform at finetyping loci (*porA*, *fetA*) [17], MLST genes (*abcZ*, *adk*, *aroE*, *fumC*, *gdh*, *pdhC*, *and pgm*) [18], ribosomal protein genes (*rpsA–rpsU*, *rplA–rplF*, *rplI–rplX*, *rpmA–rpmJ*) [19], and MenB vaccine antigen genes (*nhba*, *nadA*, *and fHbp*) [20]. Based on the allelic profile of MLST genes and ribosomal protein genes, the isolates were assigned to a sequence type (ST), clonal complex (cc) and ribosomal sequence type (rST). New gene and peptide variants were scanned manually, and after curator approval and annotation, were added to the PubMLST database. New STs and rSTs were added to the database in the same way. Genomes were compared using the BIGSdb Genome Comparator tool with the core genome cgMLST scheme v1.0 for *N*. *meningitidis* (1605 loci) [21]. Incomplete loci (due to contig breaks) were ignored in pairwise comparisons in the distance matrix calculations. The distance matrices, which are based on the number and allelic variability of the genes contained in individual schemes, were generated automatically and phylogenetic networks constructed using the SplitsTree4 software using the NeighborNet algorithm [22]. The phylogenetic networks were then edited with the graphical Inkscape tool (www.inkscape.org/en/). The studied isolates were colour-coded on the phylogenetic networks according to the observed periods, and thus it was possible to evaluate changes in the distribution of the individual genetic lines over time.

### BAST type and MenDeVAR index

The combination of peptide variants of two variable regions of the PorA protein (VR1 and VR2) and peptide variants of three antigens of MenB vaccines (NHBA, NadA and FHbp) defined the Bexsero Antigen Sequence Type (BAST) in the isolates [23]. From this, the theoretical coverage of a given isolate by both MenB vaccines was determined using the Meningococcal Deduced Vaccine Antigen Reactivity Index (MenDeVAR). This index is based on a combination of information on the presence of individual antigenic variants and the sensitivity of the antigenic variants present to antibodies in a bactericidal test [24]. According to the MenDeVAR index (https://pubmlst.org/organisms/neisseria-spp/mendevar), the isolates are classified into four groups in relation to both MenB vaccines. Isolates containing one or more specific antigenic variants included in MenB vaccines are defined as "exact match". Isolates containing one or more antigenic variants that showed cross-reactivity in experimental studies are included in the group "cross-reactive". Isolates for which enough data have not been available on their antigenic variants are defined as "insufficient data", and those carrying only antigenic variants that did not show cross-reactivity in experimental studies are classified as "none". In our study, the isolates that were defined as "exact match" and "cross-reactive" are labelled as vaccine covered and isolates that were defined as "insufficient data" are labelled as "unpredictable". Based on observations of Muzzi *et al*. [25] that 50% of isolates defined as "unpredictable" can be considered vaccine covered, we used this value in the MenDeVAR index graphs, as in the study of Freudenburg-de Graaf *et al*. [7].

### Data availability

All published WGS assembly data are publicly available in the PubMLST database [16] and individual IDs are contained in the supplementary table (S1 Table). Some of the studied isolates were included in our other studies previously [26–28].

## Results

### Genetic variability of *N*. *meningitidis* serogroup B isolates

**Fig 1:**
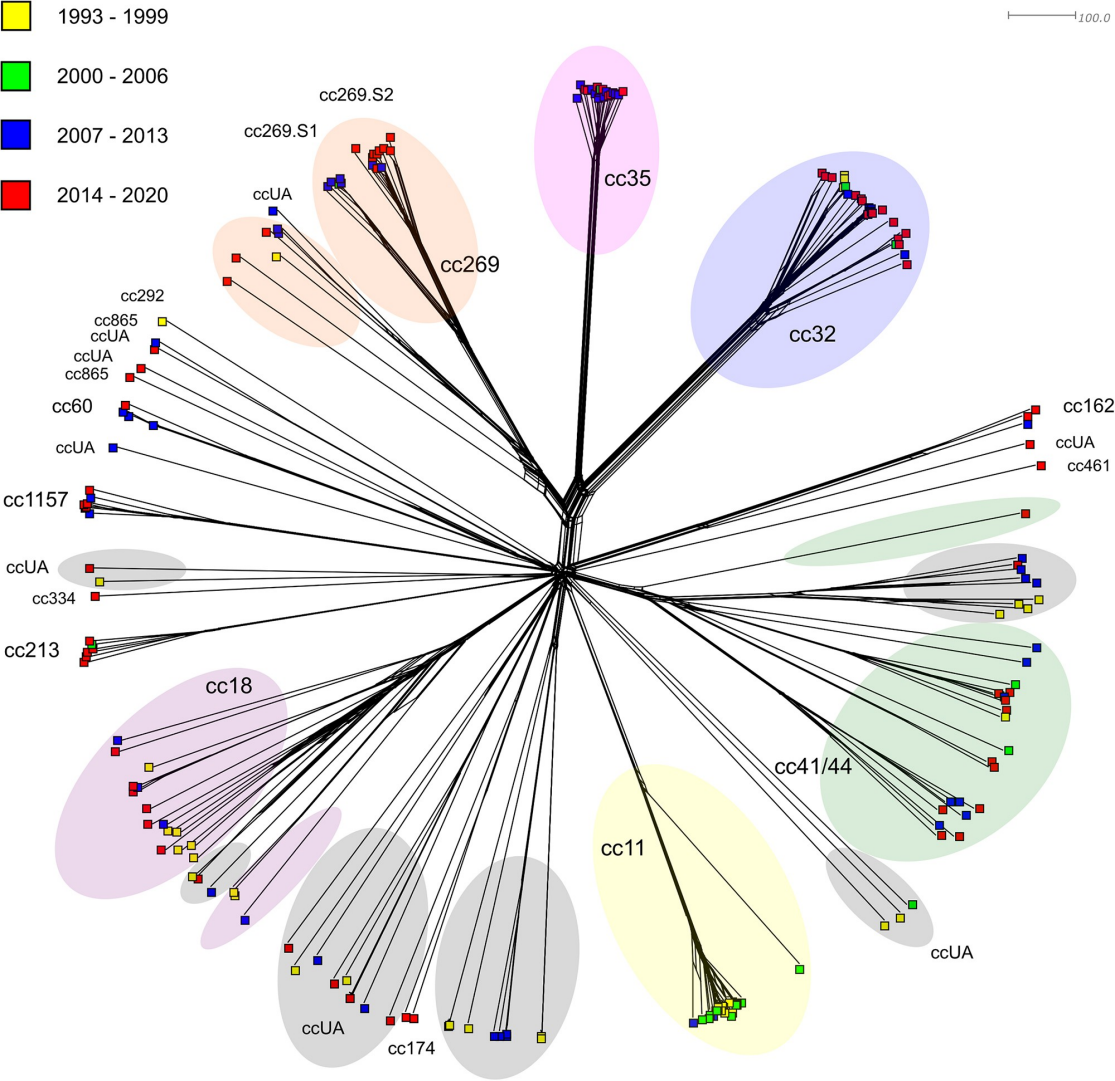
Genetic relationship of 197 MenB isolates from IMD cases collected in the Czech Republic from 1993 to 2020. Isolates are coloured according to detection year and labelled according to clonal complex.

The highest number of serogroup B isolates (MenB) belong to clonal complex cc32 (n = 26), which forms a compact but internally relatively heterogeneous cluster on the phylogenetic network (Fig 1). The isolates belong to 10 different STs. Cc32 isolates date from all the observed periods, but their frequency increased over time.

The situation is different for the highly homogeneous cluster of cc35 isolates (n = 20), which shows a distant relationship with cc32 isolates. The genotype B: P1.22–1,14:F4-1:ST-35 (cc35); rST-2548; BAST-257 was significantly dominant in the cc35 isolates, which were collected mainly between 2007–2020.

22 MenB isolates belong to complex cc269, in which we can see a distant relationship with isolates cc32 and cc35. On the phylogenetic network, the cc269 isolates form two major subpopulations. Subpopulation cc269.S1 contains 5 isolates, mainly from 2007–2013, with predominant genotype B: P1.5–1,10–4:F5-1:ST-269 (cc269); rST-2423; BAST-1102. The most common cc269 isolates at present are isolates of subpopulation cc269.S2 (n = 11), in which genotype B: P1.19–1,15–11:F1-7:ST-467 (cc269); rST-2676; BAST-222 is dominant.

Cc11 isolates (n = 23) mainly originate from the years 1993–2006. The last isolate of MenB complex cc11, which was collected in the Czech Republic, dates back to 2011. The predominant genotype is B: P1.5,2:F3-6:ST-11 (cc11) and isolate B: P1.5–1,2–2:F5-8:ST-3537 from the year 2000 stands alone.

21 isolates belong to complex cc41/44 being highly variable from a genetic point of view, which also corresponds to the highly heterogeneous molecular characteristics of isolates (14 different STs). From a temporal perspective, cc41/44 belongs to clonal complexes with a relatively constant representation, however, since 2007, an increase in the number of some lineages of these isolates within MenB has been noted.

Similar internal heterogeneity, as in the case of clonal complex cc41/44, is also shown by the isolates of complex cc18 (n = 18), in which a total of 13 different STs were determined. In the Czech Republic, cc18 isolates are among those constantly occurring in the monitored period, although this clonal complex is not among the dominant ones.

Two genetically homogeneous clusters form isolates of clonal complexes cc1157 (n = 7) and cc213 (n = 6). The dominant genotype of cluster cc1157 is B: P1.21–7,16:F5-36:ST-1157 (cc1157); rST-2933; BAST-271, and in the case of cluster cc213 (B: P1.22,14:F5-5:ST-213) we observe an increased variability in ribosomal and MenB vaccine genes. Although isolates of these complexes were rare in the past, an increase in their number (especially in cc213) was recorded in the Czech Republic in the last observed period.

### Genetic variability of *N*. *meningitidis* serogroups C, W, and Y isolates

**Fig 2:**
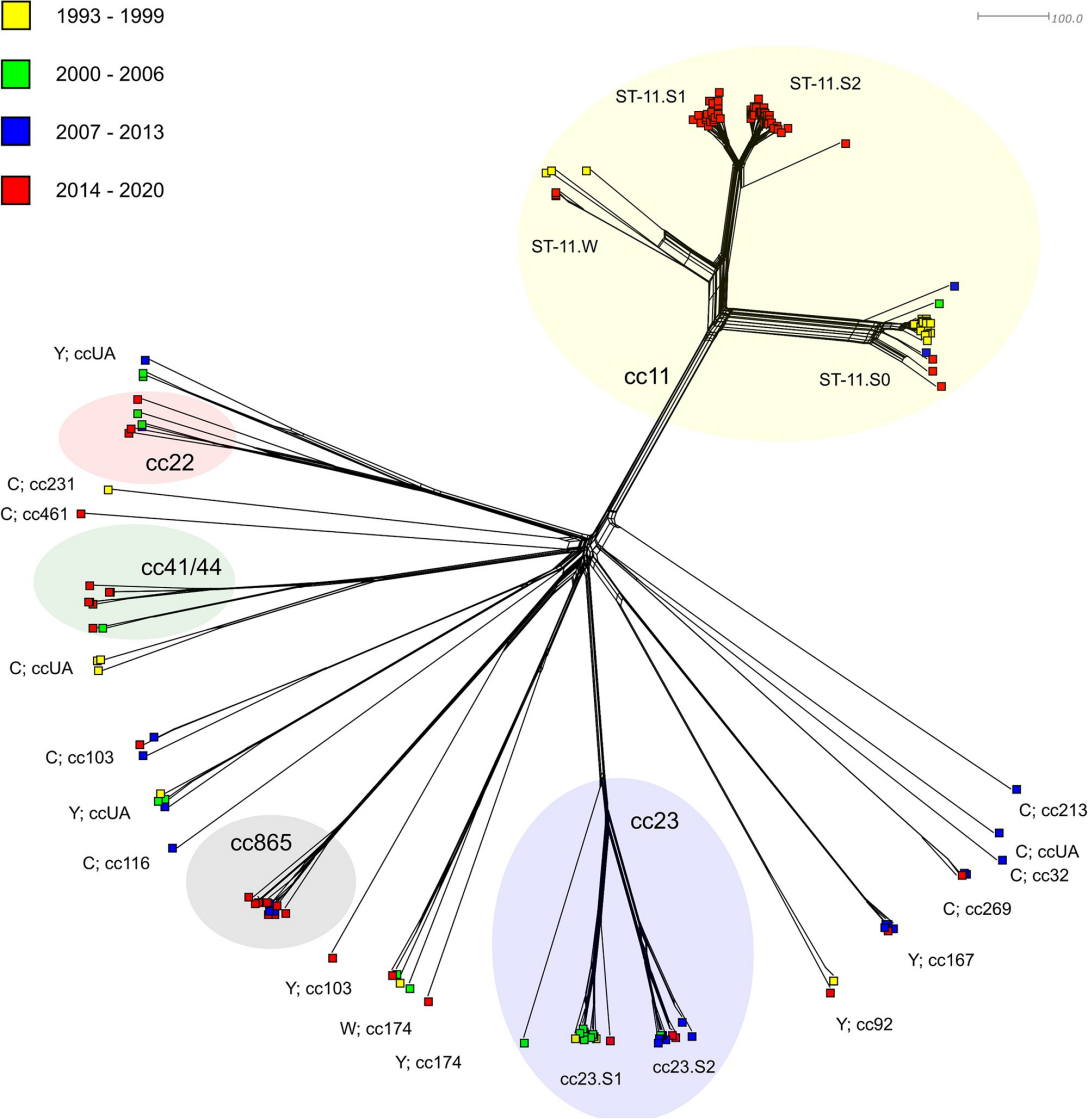
Genetic relationship of 172 non-B isolates from IMD cases collected in the Czech Republic from 1993 to 2020. Isolates are coloured according to detection year and labelled according to serogroup and clonal complex.

The phylogenetic network of serogroups C (MenC), W (MenW), and Y (MenY) consists of 172 isolates: MenC (n = 106), MenW (n = 27), MenY (n = 39). The most numerous clonal complex is cc11 (n = 89), which contains MenC (n = 83) and MenW (n = 6) isolates, which form three main genetic lineages on the phylogenetic network (Fig 2). The first lineage ST-11.S0 is made up exclusively of MenC isolates and is dominated by a cluster of isolates from 1993–1999, in which genotype C: P1.5,2:F3-6:ST-11 (cc11); rST-2343; BAST-2907 is predominant. The second genetic lineage (ST-11.W) is formed by six MenW isolates: three isolates with a dominant genotype W: P1.5,2:F3-1:ST-11 (cc11); rST-2327; BAST-3, and three isolates from the last observation period in which the predominant genotype is W: P1.5,2:F1-1:ST-11 (cc11); rST-2327; BAST-2. The last genetic lineage of clonal complex cc11 consists exclusively of MenC isolates from the years 2014–2020, which are among the most common causes of IMD in the Czech Republic today. This lineage is made up of two genetically distinct subpopulations: ST-11.S1 (n = 20) with dominant genotype C: P1.5,2:F3-3:ST-11 (cc11); rST-2328; BAST-3, and more numerous ST-11.S2 (n = 36) with genotype C: P1.5,2:F3-3:ST-11 (cc11); rST-51365; BAST-8. The only isolate that is not part of the above subpopulations and lies separately within the third genetic lineage on the phylogenetic network is isolate C: P1.5,2:F3-3:ST-5752 (cc11); rST-2328; BAST-38 from the year 2015.

21 MenY isolates belong to clonal complex cc23, which forms two genetically relatively distant subpopulations on the phylogenetic network. Subpopulation cc23.S1 mainly contains isolates from the period of 1993–2006 with dominant genotype Y: P1.5–1,2–2:F5-8:ST-569/1625 (cc23); rST-2422; BAST-227 (n = 9). The second subpopulation (cc23.S2) contains mostly isolates from the period of 2007–2020 (n = 10). In the cc23.S2 subpopulation, genotype Y: P1.5–2,10–1:F4-1:ST-23 (cc23); rST-2421; BAST-228 is predominant. The last isolate cc23, which is the only one not belonging to any of the main subpopulations, is isolate Y: P1.7–2,13–2:F4-17:ST-23 from the year 2002.

MenW cluster of isolates of clonal complex cc865, which is exclusive to the Czech Republic, contains 11 isolates mainly from the last monitored period (2014–2020). Isolates of this complex are the most common cause of IMD caused by MenW in the Czech Republic in modern times. In an initially homogeneous cluster of genotype W: P1.5–2,10–1:F5-8:ST-3342 (cc865) genetic variability increases over time, which can be seen in the increasing number of different ribosomal profiles (rST-7713/89819/89824/89825/89830/ 163663/187908). An identical change in the BAST profile was also noted in three isolates—the original BAST-1320 was changed by inactivation of the *nadA* gene allele to a newly described BAST-2939.

8 MenC isolates belong to the heterogeneous clonal complex cc41/44, which is more typical for MenB. However, an increasing incidence of MenC isolates, cc41/44, has been recorded in the Czech Republic. Following MenW cc865 isolates, cc22 is currently the second most commonly determined complex within serogroup W in the Czech Republic. The phylogenetic network shows a considerable genetic variability among the six MenW cc22 isolates from 2000–2020, for which 6 different STs and rSTs were determined.

**Fig 3:**
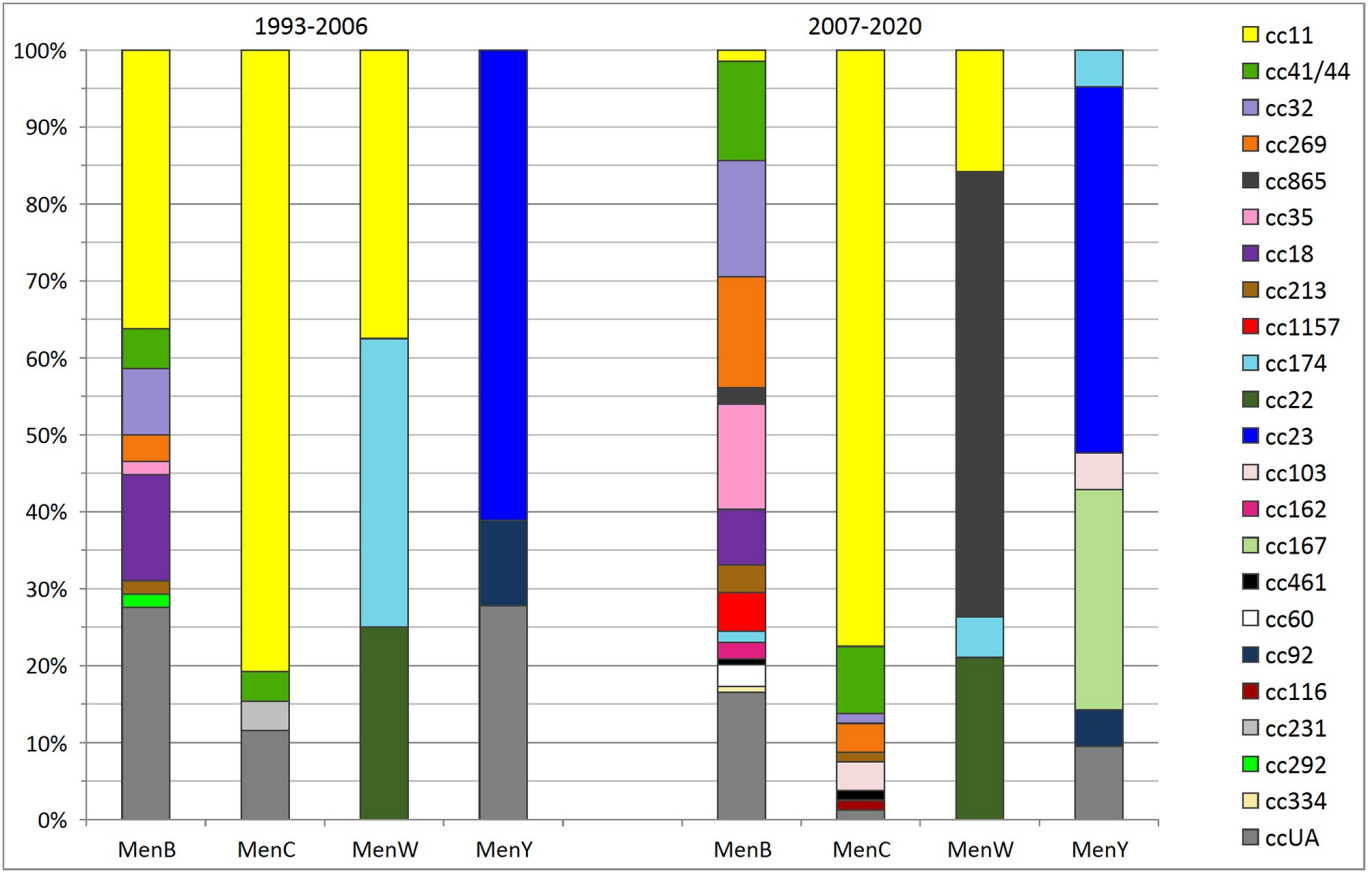
Clonal complexes of MenB, MenC, MenW, and MenY isolates from IMD cases, Czech Republic, 1993–2006 and 2007–2020.

Increasing heterogeneity (especially in MenB isolates), dominance of cc11 in MenC isolates and a high proportion of the specific cc865 complex for MenW isolates in the period 2007–2020 is evident from a comparison, that shows the representation of clonal complexes in the studied isolates from the first two (1993–2006) and the last two (2007–2020) monitored periods (Fig 3). For MenY isolates, a constant predominance of cc23 complex was observed. In the period 2007–2020, a significant proportion of MenY, cc167 (n = 6) was detected.

### Predicted coverage of *N*. *meningitidis* isolates by MenB vaccines

**Fig 4:**
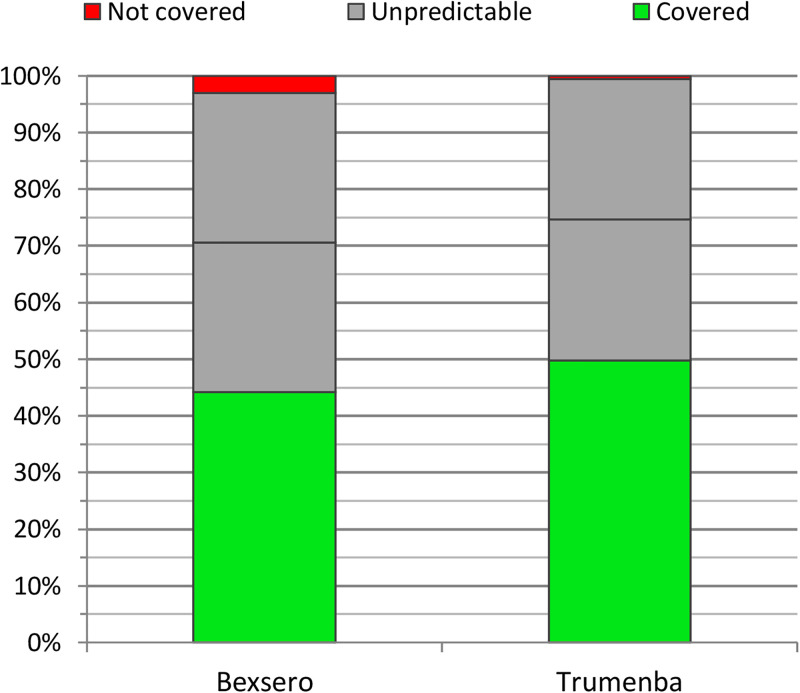
Theoretical coverage of MenB vaccines in the studied set of 197 MenB isolates from the Czech Republic, 1993–2020. Horizontal black line in the category "unpredictable" indicates 50% of the isolates in this category.

The coverage by Bexsero vaccine was determined in 87 out of the 197 (44.2%) MenB isolates by means of the MenDeVAR index, 6 isolates (3%) were marked as not covered, and the remaining 104 isolates (52.8%) carried variants of MenB antigen genes, for which data were not available in the PubMLST database presently (Fig 4). For the Trumenba vaccine, the coverage was determined for 98 isolates (49.8%), 1 isolate was defined as not covered by the vaccine, and for 98 isolates data were missing.

**Fig 5:**
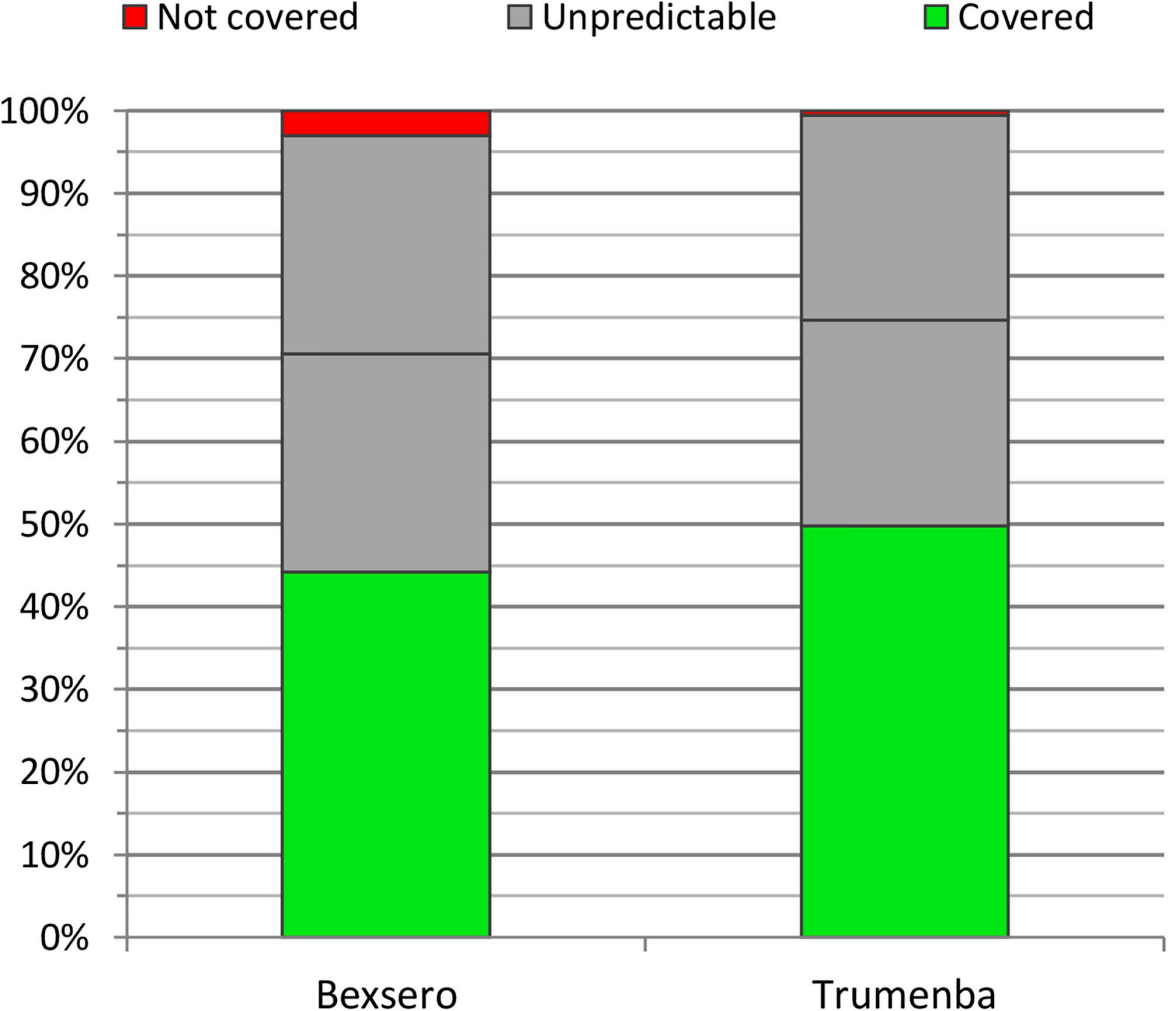
Theoretical coverage of MenB vaccines in the studied set of 172 non-B isolates from the Czech Republic, 1993–2020. Horizontal black line in the category "unpredictable" indicates 50% of the isolates in this category.

For MenC, MenW, and MenY isolates, out of the total of 172, based on the MenDeVAR index, 43 (25%) were defined as covered by Bexsero vaccine, 1 isolate was defined as not covered, and for the remaining 128 isolates (74.4%), data were not yet available (Fig 5). There were 53 (30.8%) isolates that were defined as covered by Trumenba, and for the remaining 119 (69.2%) data were missing.

If half of the isolates for which MenDeVAR index data are missing from the PubMLST database were covered by the vaccines, the theoretical Bexsero vaccine coverage would be 70.6% (for MenB) and 62.2% (for MenC, W, Y). For the Trumenba vaccine, under the same assumptions, the theoretical coverage would be 74.6% (for MenB) and 65.7% (for MenC, W, Y).

## Discussion

MenB isolates, which were collected in the Czech Republic between 1993 and 2020, can be summarized as a heterogeneous population, in which heterogeneity continues to increase over time. The most abundantly represented were 8 different clonal complexes, some of which showed constant representation during all four monitored periods (cc18, cc35, cc41/44), a gradual increase in frequency was recorded for other clonal complexes (cc32, cc213, cc269, cc1157). An increase in complex cc213 in MenB isolates from IMD was also found in a Spanish study, which reported an increase from 3.6% in 2007 to 33% in 2018 [29]. On the other hand, in the case of the previously frequent complex cc11, the frequency decreased to a complete absence in the Czech Republic in the last monitored period (2014–2020). Isolates of less frequent complexes (cc60, cc162, cc174, cc292, cc334, cc461, cc865) occurred sporadically, while isolates of MenB unassigned to a clonal complex (ccUA) were highly represented in the studied set. The internal heterogeneity of individual complexes was also highly variable—from homogeneous clonal complexes (cc35, cc213, cc1157) to highly heterogeneous clonal complexes (cc18, cc41/44). The high heterogeneity of MenB isolates from IMD was also demonstrated in a Dutch study from 2017–2019, where 11 different clonal complexes were detected, the most common of which were: cc32, cc41/44, cc213 and cc269 [7]. A study from the Republic of Ireland over a 19-year period (1996–2015) also demonstrated a high heterogeneity of MenB isolates from IMD. During the monitored years, a decrease in cc32 and cc41/44 and, conversely, an increase in cc269 and cc461 were recorded [30].

Among MenC, MenW, and MenY isolates, the heterogeneity was significantly lower than that of serogroup B. In the case of MenC, isolates of clonal complex cc11 dominated, forming two main lineages. The first lineage contained mostly isolates from 1993–1999 and the second was composed exclusively of isolates from 2014–2020, which clustered into two subpopulations. With the exception of complex cc41/44, which currently shows an increasing frequency, the numbers of MenC isolates of the other clonal complexes were minimal throughout the observed period. A similar situation, i.e. the predominance of the hypervirulent clonal complex cc11 in MenC isolates from IMD, has been observed in a number of studies [31–33]. Phylogenetic analyses have shown that the hypervirulent strain C, cc11 (UK-strain) has spread in a number of European countries [34]. A study from the Republic of Ireland over a 19-year period (1996–2015) also demonstrated the homogeneity of MenC isolates from IMD, with a prevalence of cc8 and cc11 [30].

The highest number of MenW isolates belonged to clonal complex cc865, which we described as specific exclusively for the Czech Republic [26]. The unusual sequence type ST-3342 (cc865) in association with serogroup W was detected only in the Czech Republic. In relation to serogroup B, ST-3342 was recorded in isolates from 2010–2013 in the Czech Republic (n = 4) and Poland (n = 3) according to the international database PubMLST [16]. These facts, together with the position of this cluster on the phylogenetic network, support the theory that the subpopulation of isolates MenW, cc865 originated in the Czech Republic from MenB isolates by a capsule switching mechanism. MenW cc865 isolates were detected in the Czech Republic only in the last two monitored periods (2007–2020), and a gradual increase in their genetic variability was noted in this initially homogeneous cluster. The remaining MenW isolates belonged to three different clonal complexes (cc11, cc22, cc174). The emergence of a new genetic lineage ST-9316 in MenW isolates from IMD was also noted in France in 2013–2018 [35]. The mechanism of capsule switching is also observed in other serogroups of *N*. *meningitidis*, for example, in Italy, an outbreak of IMD was caused by the hypervirulent B strain, cc11, which, according to a WGS data analysis, probably originated from the hypervirulent C strain, cc11 [36]. In clonal complex cc11, the mechanism of capsule switching from MenC to MenB is frequently observed and has the potential to cause high morbidity and mortality in IMD [37].

A dominant clonal complex of MenY isolates was cc23, which formed two genetically quite distant subpopulations and which showed constant representation throughout the observed period. A similar situation, i.e. predominance of cc23 in MenY isolates from IMD, has been reported in a number of studies [38–40]. Apart from cc167 isolates from period 2007–2020 and ccUA isolates, within serogroup Y, isolates of other clonal complexes were collected only exceptionally (cc92, cc103, cc174).

Both MenB vaccines are being studied globally for their potential to cover non-B isolates from IMD. In a European and Brazilian panel of 147 MenC, MenW, and MenY isolates, 74% demonstrated a bactericidal effect induced by Bexsero vaccine [41]. The potential of this vaccine to reduce the incidence of IMD caused by MenW meningococci was also demonstrated in IMD surveillance in England [6]. In our study, a theoretical MenB vaccine coverage for both MenB and non-B isolates was determined based on the MenDeVAR index. The theoretical efficacy of Bexsero vaccine was 70.6% for MenB isolates and 62.2% for MenC, W and Y isolates in the examined set. For Trumenba, the values of theoretically covered isolates were 74.6% for MenB isolates and 65.7% for MenC, W and Y isolates. Given that the MenDeVAR index was based on MenB isolates assays [24], our estimations of coverage for MenC, W and Y isolates may have limitations.

The results of the WGS data analysis demonstrated increasing heterogeneity (especially in MenB isolates), dominance of cc11 in MenC isolates over the entire observed period, and a high proportion of the specific cc865 complex for MenW isolates in the period 2007–2020. Our results have shown sufficient coverage of Czech heterogeneous population of *N*. *meningitidis* with MenB vaccines and, together with surveillance data on invasive meningococcal disease in the Czech Republic, were the basis for updating recommendations for vaccination against invasive meningococcal disease. It is necessary to continue further study so that the vaccination strategy in the Czech Republic corresponds to the epidemiological situation.

## Supporting information

S1 Table PubMLST IDs of 369 isolates of *N*. *meningitidis*, Czech Republic, 1993–2020. (XLSX)
